# Prevalence of HPV 16 and 18 and attitudes toward HPV vaccination trials in patients with cervical cancer in Mali

**DOI:** 10.1371/journal.pone.0172661

**Published:** 2017-02-23

**Authors:** Ibrahima Téguété, Amadou Dolo, Kotou Sangare, Abdoulaye Sissoko, Mali Rochas, Sarah Beseme, Karamoko Tounkara, Shahla Yekta, Anne S. De Groot, Ousmane A. Koita

**Affiliations:** 1 Hôpital Gabriel Touré, Bamako, Mali; 2 Laboratory of Applied Molecular Biology (LBMA), Science and Technologies Faculty (FST), University of Science Techniques and Technologies of Bamako (USTTB), Bamako, Mali; 3 GAIA Vaccine Foundation, Providence, RI, United States of America; 4 Foundation GAIA, Bamako, Mali; 5 Institute for Immunology and Informatics (iCubed), University of Rhode Island, Providence, RI, United States of America; Fondazione IRCCS Istituto Nazionale dei Tumori, ITALY

## Abstract

**Background:**

Cervical cancer is one of the most common and lethal cancers in West Africa. Even though vaccines that protect against the most common Human papillomavirus (HPV) strains, 16 and 18, are currently in use in developed countries, the implementation of these vaccines in developing countries has been painfully slow, considering the pre-eminence of HPV-associated cervical cancer among women in those countries.

**Aim:**

We performed serological and PCR-based assessment of blood and tissue specimens obtained from women undergoing cervical cancer-related surgery at a major urban hospital in Bamako. Since several therapeutic HPV vaccines are currently in clinical trials, we also assessed willingness to participate in HPV cancer vaccine trials.

**Methods:**

Blood and biopsy samples of 240 women were evaluated for HPV types 16 and 18 by serology and PCR. Knowledge regarding the HPV vaccine and autonomy to decide to vaccinate their own child was assessed with a standardized questionnaire.

**Results:**

HPV 16 and 18 were identified in 137/166 (82.5%) cervical cancer biopsy samples by PCR. Co-infection with both HPV 16 and 18 was significantly more frequent in women over 50 years of age than in younger women (63.0% vs. 37.0%). 44% of study participants said they would be willing to vaccinate their child with HPV vaccine. Only 39% of women participating in this study reported that they would be able to make an autonomous decision to receive HPV vaccination. Permission from a male spouse or head of household was identified as important for participation by 59% of the women.

**Conclusion:**

This study provides strong support for the introduction of currently available HPV vaccines in Mali, and also provides key information about conditions for obtaining informed consent for HPV vaccine trials and HPV vaccination in Mali.

## Introduction

Nearly 85% of the half a million cervical cancer (CC) cases diagnosed each year occur in developing world countries. There are 27,300 new cases of CC identified in West Africa every year, and 16,500 deaths per year are attributed to CC [1]; CC is the second most common and lethal cancer among women in the region. Mali, a landlocked sub-Saharan West African country has an estimated population of 4.04 million women aged 15 and over, and the highest regional rate of CC (44.2 cases of CC per 100,000 women [2]. Cervical cancer is the leading cause of all cancer-related mortalities among women aged 15 to 44 years in Mali. Sixty-eight percent of women diagnosed with CC in Mali do not survive, an equivalent of 1,261 deaths per year [3]. The high mortality rate is likely due to the low accessibility of CC screening: according to household surveys performed by WHO, only 4% of women have ever been screened for CC [3]. The mortality rate from cervical cancer may actually be higher than reported here, as many residents of Mali are unable to afford medical care (78% of the population earned less than $3.10 per day in 2009 [4]) and thus cancer cases are likely to be under-reported.

The main risk factor of CC is infection with high-risk types of human papillomavirus (HPV) [5]. As defined by the World Health Organization (WHO), there are 12 high-risk cancer-causing HPV types [6]. Among them, HPV types 16 and 18 are found in 60% to 70% of invasive cervical cancer (ICC) cases in Africa [7–10]. The prevalence of high-risk types is lower among women who have not been diagnosed with cervical cancer. The prevalence of high-risk types has been estimated to be 12% among Malian women aged 15–65 [11,12] and was reported to be 23% in urban women living in Bamako, Mali in 2013 [13]. Prevention of HPV infection by prophylactic vaccines may address this major public health concern. Therapeutic vaccines for the treatment of cervical cancer are in clinical trials [14,15] these vaccines primarily focus on high-risk types HPV 16 and 18. Thus it is important to determine the usefulness of available HPV vaccines by evaluating the prevalence of high-risk HPV types in cervical cancer patients.

The most recent HPV typing data trace back to 1995 and 2002 which showed that high-risk HPV types 16 and 18 could be identified in 54% to 62% of CC cases in Mali [16,17]. To address the lack of recent data on the prevalence of high-risk HPV types among women being treated for cervical cancer in Mali, we evaluated the prevalence of HPV subtypes 16 and 18 among women who had histologically confirmed diagnoses of CC at Hôpital Gabriel Touré in Bamako, Mali by PCR. We also assessed the knowledge, attitudes and practices regarding vaccination and determined willingness to participate in a clinical trial of an HPV vaccine.

## Methods

### Recruitment of participants

Hôpital Gabriel Touré has an annual admissions rate of about 16,500 patients and serves two million patients in the direct community surrounding the hospital (Bamako proper) and 12 million in total across the wider region. It is the primary referral hospital for cervical cancer in Mali and 95% of cervical cancer patients diagnosed in Bamako are referred to this hospital.

During the study period more than 3,344 patients were seen in the department of gynecology, of which 10% were diagnosed with cervical cancer (roughly 334). This study was performed over 20 months during that period and captured nearly 88% of the patients who had cervical cancer confirmed by biopsy during that period.

From June 2011 to January 2013, all patients with histologically confirmed CC were approached by clinicians at the Gabriel Touré Hospital and asked whether they would be willing to participate in a study of HPV infection and in a survey. 240 women agreed to participate; informed consent was obtained for their participation. No compensation was offered in exchange for participation in the study. 240 women consented to the use of their cervical biopsy specimen and provided blood for serology; 235 women answered the survey questions (five declined to participate in the survey portion of the study). Cervical biopsy specimens and serum samples were processed at the Laboratory of Applied Molecular Biology at the University of Bamako.

### Collection of general and medical information and knowledge and attitude toward vaccination

A standardized questionnaire was read to the participants in order to collect general and medical information and willingness to participate (WTP) in an HPV vaccination program and vaccination decision-making capacity. The questionnaire used in this study was adapted from a pilot study published by our group in 2013 [18]. The structured interviews were conducted in Bambara, the primary language in Mali, by trained medical staff. An English translation of the survey document is provided in the supporting information file (see S1 File).

### Sample collection

Whole blood samples (4 mL) were collected and centrifuged at 4000 rpm for 20 minutes; serum samples were transferred in cryovials, stored at -80°C and sent to an immunology laboratory (PPD Vaccines and Biologics Lab, Wayne, PA) for serological marker detection of HPV subtypes 16 and 18 (see below). Cervical biopsies were collected in 400 μL of 10X Dulbecco's Phosphate-Buffered Saline (PBS, Life Technologies, Grand Island, NY) and stored at -80°C for further examination by polymerase chain reaction (PCR) assay for detection of HPV 16 and 18 DNA.

### DNA extraction and HPV detection by PCR

Total DNA was extracted from biopsies using the QIAamp DNA Blood Mini Kit (Qiagen, Valencia, CA) following the manufacturer's instructions: 15–25 mg tissue sections were applied to a clean single-edge blade using strict aseptic technique to prevent contamination or cross-contamination of samples. The samples were digested overnight at 55°C in the lysis Buffer AL containing protease K (Qiagen, Valencia, CA), followed by precipitation of DNA with absolute ethanol in a spin column. The DNA was eluted with Buffer AE (Qiagen, Valencia, CA). DNA purity and concentration were quantified using a BioPhotometer (Eppendorf, Hauppauge, NY). Two sets of primers specific to the E6 oncogene were used to detect HPV types 16 and 18 DNA (p16F/p16R and p18F/p18R, respectively, Table 1) [19–21]. The expected size for both PCR products was 140 bp. DNA integrity was determined by amplification of the human β-globin gene prior to HPV PCR assay, as described in the literature [19,22] using Globin-1 and -2 primers (Table 1). The expected size for the PCR product was 268 bp.

**Table 1:** Primer sequences.

Primer Name	Sequence (5’-3’)
p16F	AAGGGCGTAACCGAAATCGGT
p16R	GTTTGCAGCTCTGTGCATA
p18F	AAGGGAGTAACCGAAAACGGT
p18R	GTGTTCAGTTCCGTGCACA
Globin-1	GAA GAG CCA AGG ACA GGT AC
Globin-2	CAACTTCATCCACGTTACACC

The PCR assays were performed as described by others [21,22] with minor modifications. All reagents were from Life Technologies, Carlsbad, CA, unless otherwise specified. Briefly, test sample DNA (10 to 200 ng) was added to a mixture containing dNTP (0.2 mM each), Taq polymerase (0.04 U), reaction buffer (1X), MgCl_2_ (1.5 mM), reverse and forward primers for HPV 16 or HPV 18 (0.5 μM each; Eurofins MWG, Les Ulis, France); PCR-quality water was used to reach a final volume of 25 μL. To avoid false positive and/or negative results, a negative control (no template DNA) and an HPV-positive DNA sample were included in each assay. PCR amplification was performed using a DNA Thermocycler (MJ Research, Waltham, MA, USA), as follows: 5 min at 94°C, 40 cycles (94°C, 1 min; 65°C, 1 min; 72°C, 2 min) and a final 7 min extension period at 72°C.

As a control of the PCR product size, 12 μl of PCR product were loaded on a 2% 0.5X TBE buffer agarose gel, stained with 0.3 mg/mL ethidium bromide, and photographed by UV transillumination with a KODAK EDAS 290. The molecular weight of the PCR products was determined by comparison to DNA molecular weight marker XIV (Roche Diagnostics, Mannheim, Germany).

### Detection of HPV antibodies by cLIA assays

The presence of antibodies to HPV types 16 and 18 was detected by a VLP-based competitive Luminex immunoassay (cLIA) using a multiplexed Laboratory Multi-Analyte Profiling (LabMAP3) assay system (Luminex Corporation, Austin, TX) as previously described [23,24]. Briefly, HPV VLPs 16 and 18 (Merck & Co., Whitehouse Station, NJ, USA) were covalently coupled to fluorescent microsphere beads (Luminex Corporation, Austin, TX) according to the manufacturer’s instructions and stored at a concentration of 1.25x10^7^ microspheres/mL in the dark at 4°C. A total of 5,000 of each VLP16- and VLP18-microspheres were combined with 50 μl of 1:4 diluted blood samples in a dark 96-well plate. To each well, 25 μl of HPV type-specific mAbs conjugated to PE (Merck & Co., Whitehouse Station, NJ, USA) were added, followed by an overnight incubation at room temperature. Samples were transferred to a filter plate (Millipore, Bedford, MA) and washed in PBS; the VLP-microspheres were re-suspended in 200 μl of PBS containing 1% BSA for analysis on a BioPlex Suspension Array System (BioRad, Hercules, CA).

The reference standard used for analysis was a serial two-fold dilution of a pool of serum samples from African green monkeys hyperimmunized with monovalent HPV L1 VLP types 16 and 18. Dilution-corrected serum values (in mMU/mL) were computed based on a four-parameter logistics fit of the standard curve on each assay plate. Samples that exceeded the limits of quantitation for the standard curve were retested at higher dilutions. Samples were considered Ab seropositive for the respective HPV subtype if the cLIA signal was ≥11 mMU for HPV 16 and ≥10 mMU for HPV 18. All standards, controls, and samples were tested in duplicate.

### Statistical analysis

GraphPad (http://www.graphpad.com) was used to calculate 95% confidence intervals. The Fisher test was applied to determine significant differences between groups. p<0.01 was considered significant.

### Ethics statement

The study protocols for obtaining informed consent, tissue samples, blood tests, and surveys were reviewed and approved by the Committee of Ethics of the Faculty of Medicine, Pharmacy and Odonto-stomatology (FMPOS) in Bamako, Mali. The informed consent document was translated from French to Bambara. The consent form was read aloud to individuals who were illiterate. All participants provided a signature or fingerprint indicating their informed consent. All participants were over 18 years old.

## Results

### Characteristics of women diagnosed with CC

Between June 2011 and January 2013, all of the patients undergoing biopsy for cervical cancer at Hôpital Touré, the primary referral hospital for cervical cancer in the region of Bamako, were approached to participate in this study. 88% (240) of the more than 270 women who had biopsies during that period agreed to have their serum obtained and their biopsy specimen evaluated for HPV testing. Of these 240 women, 235 agreed to participate in the structured interview.

Characteristics of the study participants are summarized in Table 2. The mean age of the participants was 50.0 years (SD: 13.2); with 133 (56.6%) of the participants aged 20–50 years and 102 (43.4%) aged over 51 years. Of the 231 (98.3%) participants who were married (including divorced and widows), 51 (22.1%) reported having been in polygamous marriages. The mean age of first marriage and when the first child was born was 17.0 years (SD: 2.7 and 2.3, respectively) and the mean age for sexual debut was 16.0 years (SD: 2.1). Among the 229 women with children, the average number of children was 6 (SD: 2.9). Only 40 patients (17.0%) had attended either primary or secondary school and 27 (11.5%) had been employed outside their home.

**Table 2:** Characteristics of the 235 women who agreed to participate in the structured interview.

		N (235)	%
**Characteristics**			
Mean Age (SD)		50.0 (13.2)	
	Age (years) <29	14	6.0
	30–49	119	50.6
	>50	102	43.4
Married		231	98.3
	Polygamous marriage*	51	22.1
	Mean age of first marriage (SD)	17.0 (2.7)	
Mean age of sexual debut (SD)		16.0 (2.1)	
Average number of children (SD)^†^		6.0 (2.9)	
Mean age when first child born (SD)^†^		17.0 (2.3)	
Ever attended school		40	17.0
Employed outside the home		27	11.5
**Medical history**			
Age of CC diagnosis (SD)		49.0 (13.6)	
	<29 years	18	7.7
	30–49 years	124	52.8
	>50 years	92	39.1
Known history of STI		134	57.0
	Pruritus, leucorrhea^‡^	47	35.1
	Other	73	54.5

Notes

* Among those who were married; N = 231.

† Among those with children; N = 229.

‡ Among those with a history of STIs; N = 134

The mean age of CC diagnosis was 49.0 years (SD: 13.6): 142 participants (60.4%) were diagnosed between the ages of 20–50 years, and 92 (39.1%) were diagnosed between 50–90 years. 134 participants (57.0%) reported having previous sexually transmitted infections (STIs).

### Prevalence of HPV 16 and HPV 18 infection

**Fig 1:**
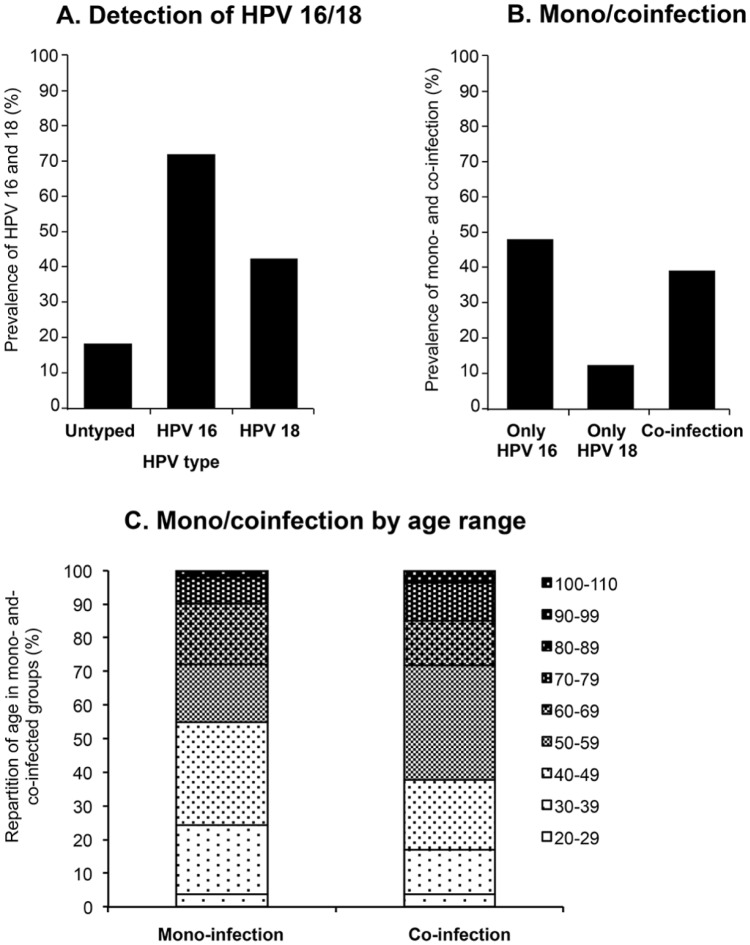
Prevalence of HPV 16 and 18 in women with cervical cancer. **A**. Percentage of positive samples for HPV 16 and 18 by PCR (n = 166). Untyped samples were negative for both HPV 16 and 18. **B**. Percentage of positive samples for HPV 16 only, HPV 18 only (mono-infection) or both HPV 16 and 18 (co-infection) by PCR (n = 135). **C**. Repartition into age groups of positive samples for HPV 16 or HPV 18 (mono-infection) or for both (co-infection) by PCR. For mono- and co-infections, lighter shades represent the percentage of infected women within the co- or mono-infected group aged 49 years or younger; darker shades represent the percentage of infected women aged 50 years or older.

Cervical biopsy samples (N = 240) were tested by PCR using human β-globin pair primers as a control for sample integrity; 166 (69.2%) were PCR-positive for human β-globin. Of these 166 samples, 31 specimens were not positive for either genotype (18.7%; 95% CI: 13.3–25.0) (Fig 1A). For the remaining 135 samples, HPV 16 genotype was detected in 118 (71.1%; 95% CI: 64.2–77.7) and HPV 18 was detected in 70 (42.2%; 95% CI: 35.1–49.8) (Fig 1A). 31.9% of the biopsy specimens were positive for both HPV 16 and 18. The primers used in this experiment were designed to amplify only HPV 16 and 18 subtypes, thus the negative samples were classified as “untyped” and are presumed to be HPV-positive for non-16 or 18 subtypes, or false negative by PCR assay.

### Prevalence of HPV 16/18 co-infection

In samples where HPV 16 and/or 18 was detected (n = 135), mono-infection with HPV 16 or HPV 18 was found in 65 (48.2%, 95% CI: 39.9–56.5) and 17 (12.6%; 95% CI: 7.9–19.3) cervical biopsy samples, respectively (Fig 1B). Co-infection with both HPV 16 and 18 was detected in 53 samples (39.3%; 95% CI: 31.4–47.7).

The age distributions of mono- and co-infected women were different (Fig 1C). Significantly fewer women aged 30–49 years old were co-infected (N = 18/53; 34.0%; 95% CI: 22.6–47.5) than women over 50 years old (N = 33/53; 62.3%; 95% CI: 48.8–74.1). No difference was observed for women under 29 years old, nor was any difference found for the mono-infected women 30–49 years old and over 50 years old (N = 42/82; 51.2%; 95% CI: 40.6–64.7 vs. N = 37/82; 45.1%; 95% CI: 34.8–55.9). These results may suggest that continued exposure over time, with age, can be a risk factor for increased co-infection with high-risk HPV 16 and 18 subtypes among older women diagnosed with CC.

### Presence of neutralizing antibodies to HPV 16 and 18

**Fig 2:**
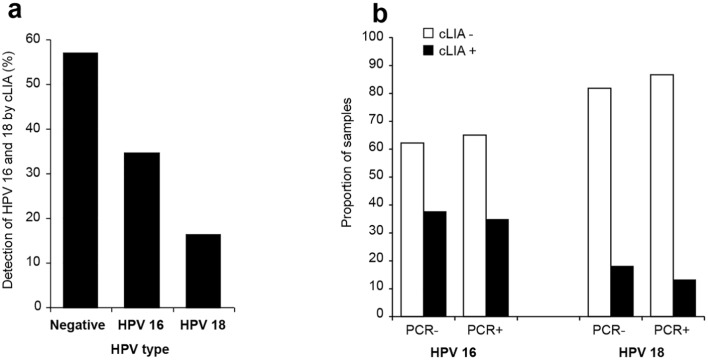
Detection of HPV16/18 neutralizing antibodies. Presence of antibodies to HPV types 16 and 18 by a VLP-based competitive Luminex immunoassay (cLIA). Results are presented as percentage of total number of available blood samples (N = 160).

Another means to detect HPV infection (or the efficacy of HPV vaccination) is to measure the presence of anti-VLP-16 and -18 antibodies in the serum. Blood was obtained from 160 of the 240 patients; serum antibodies were assessed using a cLIA assay to measure the presence of anti-VLP 16 and 18 antibodies in the serum. HPV 16 antibody-positive blood samples were more frequent (N = 56; 35.0%; 95% CI: 28.0–42.7) than HPV 18 (N = 27; 16.9%; 95% CI: 10.5–25.9) (Fig 2), which was consistent with the PCR results from this study. However, the proportion of untyped samples was higher using cLIA than PCR (18.5% by PCR vs. 57.5%, 95% CI: 49.8–64.9 by cLIA).

### Willingness to participate and autonomy to participate in an HPV vaccine trial

**Fig 3:**
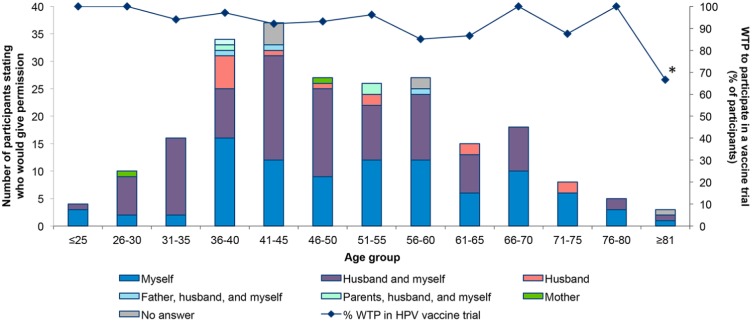
Willingness to participate and who would give permission for participation in a HPV vaccine trial. The percentage of women willing to participate in a HPV vaccine trial is represented by age group with the line. The bars represent the number of women answering the question who would give permission to participate to a HPV vaccine trial per age group. The answers were myself only (dark blue), husband and myself (purple), husband only (pink), father, husband and myself (light blue), parents, husband and myself (light green), mother only (dark green), or no answer (grey). In the oldest age group, the * asterix indicates data that only includes 3 patients.

235 women participated in the oral survey. Two hundred and four participants (86.8%) were able to recall that they had previously received any vaccine, and 228 (97.0%) said they would like the HPV vaccine to be available to young adolescent girls in Mali (Table 3). Nearly all participants (n = 220; 93.6%) also reported that they would be willing to participate in an HPV cancer vaccine trial (Table 3). Very few of the women reported that they would be able to autonomously decide to participate in an HPV vaccination study. The majority of women (139 or 59.1%) reported that a man (father, husband, or both) were identified as decision makers regarding HPV vaccination consent (Fig 3). Among the 214 (91.1%) women who stated that they would make the decision themselves, 121 (56.5%) said their husband would also decide. Thus, only 93 women (38.8%) reported that they, alone, could autonomously decide to be vaccinated.

**Table 3:** Attitudes about HPV vaccination and decision-making.

		N (235)	%
Vaccination			
Have been vaccinated in the past		204	86.8
Would like the HPV vaccine to be available in Mali to:			
	Adolescent girls	228	97.0
	Adolescent boys	92	39.1
	Women	150	63.8
	Men	15	6.4
Would participate in a vaccine trial		220	93.6

## Discussion

This study reveals important information on HPV prevalence and subtype distribution among women diagnosed with CC in Mali, and to our knowledge, it is the first such study conducted in Mali since 2002 [17]. This study also provides information to guide the implementation of HPV vaccines, including therapeutic HPV vaccines in West Africa and the consent process for engaging women in vaccine trials.

In our study, HPV 16 and/or 18 was detected by PCR for 81.5% of CC patients. This prevalence of the infection is consistent with the last study published in 2002 by Bayo *et al*. [17], where 96.9% of the 97 women with CC were infected with one of the 8 HPV subtypes tested. However, the prevalence of HPV subtype 16 was much higher in our study (71.1%) than in the previous study (50.0%). The same observation was made with HPV 18 (42.2% in our study vs. 12.7% in Bayo *et al*) and with co-infection (31.9% of the 166 participants in our study vs. only 11.1%). These differences may be due sample size or to differences in the methods used; in the Bayo et al study, the authors used the PCR enzyme immunoassay method [25] while we performed a PCR using specific primers [21]. Studies in Sub-Saharan African countries have demonstrated that HPV 16 and HPV 18 contribute to 68% of cervical cancers [26]. We found that HPV 16 and 18 were even more common in this study (confirmed in 82.5% of cervical cancer patients) however we did not identify the HPV type in 17.5% of subjects. Those subjects may have had HPV 45, or other types (HPV 35, HPV 33, HPV 52) that have been reported to be more common in Sub-Saharan Africa [27]. The sample size may not be sufficient to give the actual distribution of less common HPV subtypes in Mali.

The mortality rate of cervical cancer in Mali is very high (69%). Vaccines that protect against HPV infection are in use in the developed world, and therapeutic vaccines are in clinical trials [14]. Our results provide support for the introduction of these vaccines in Mali, since the predominant type of HPV identified in cervical cancer specimens were shown to be one of the two types that are targeted by the prophylactic vaccines.

Co-infection has been shown to be associated with persistent HPV infection and therefore has also been associated with development of CC [28]. In a previous meta-analysis, 4–20% of women with diagnosed CC showed co-infection with HPV 16 and 18 [29]. In our study the prevalence of co-infection was higher (31.9%). A higher prevalence of co-infection (36%) has also been described among women diagnosed with HPV infection in rural Costa Rica [30]. However, our finding could be the result of a possible cross reaction between the HPV16 and HPV18 primers used in this study, as such cross reaction has been described [31]. The cLIA assay also showed that 22% of positive samples were positive for both HPV 16 and 18 antibodies (data not shown). This suggests that although co-infection rates measured by PCR in our study might be overestimated; this group of patient had a higher co-infection rate than previously described. Co-infection rates were also higher in older study participants. If rate of co-infection is linked to cervical cancer prevalence, co-infection may be one factor that contributes to the high prevalence of cervical cancer in West Africa.

As has been reported previously, serology (cLIA assay, in this study) was less sensitive than the PCR assay to detect type-specific HPV infection in cervical cancer patients. This assay is primarily used for measuring effective sero-conversion soon after HPV vaccination. Regardless, the prevalence of HPV 16 and 18 by these two methods (one or both subtypes found in 81.5% of women by PCR and 43.5% of women by the less-sensitive antibody test) indicate that these genotypes are associated with CC among Malian women.

HPV prophylactic vaccines were being introduced in Mali at the time of this study and therapeutic HPV cancer vaccines were under development. Thus, we also assessed the willingness of these women to participate in vaccine trials, and perhaps more important, their ability to autonomously give consent for participation in a clinical study. Among these participants who already were affected by cervical cancer, 93.6% were willing to participate in a CC vaccine trial. In addition, 97% of the participants agreed that the HPV vaccine should be made available to adolescent girls. This data and similar data published in a companion study [32] provide support for the introduction of preventive vaccines and suggest that acceptance would be high. Acceptance of vaccination is critically important to the success of a vaccination campaign [33].

The ability of the female participants in this study to make autonomous decisions reflects local customs. Most of the participants identified men (husbands and fathers) as the primary decision-makers regarding their participation in HPV vaccination trials. In our parallel study, we also assessed autonomy regarding vaccination [32] among community members. The results were consistent with data collected here. In both studies, men (spouses, heads of household) were also identified as the primary decision makers about whether their own children would be able to participate in clinical studies.

Several studies have shown that women’s autonomy is positively related to children’s health outcomes [34]. Since the rate of cervical cancer is so high, and cancer screening is not universally available in Mali [35], it is clear that an important strategy for the successful introduction of HPV vaccination would be to engage both men and women in education about the link between HPV and cervical cancer and the efficacy of HPV vaccination. Additional research is needed to further clarify the decision-making dynamics with regard to HPV vaccination of dependent children and to incorporate this information into public awareness messages and targeted HPV vaccine campaigns.

We also note that the women in this study reported that their age of sexual debut was 16, consistent with published studies for this region of the world [17]. Prophylactic HPV vaccination has been approved for children prior to their sexual debut, and the maximum age for vaccination is therefore set at 12 to 13 in many parts of the world. This study supports the concept that HPV vaccination could be extended to girls up to the age of 15 in Mali.

Even though cervical cancer screening is available in certain health clinics in Bamako, lack of public awareness about HPV and CC is one of the most important barriers to effective CC prevention. Additional studies by our group [32] have demonstrated that limited knowledge about the purpose and location of CC screens is a problem that must be addressed in order to achieve greater HPV and CC prevention.

In conclusion, this study provides important information on HPV prevalence and subtype distribution among women diagnosed with CC in Mali, and to our knowledge, it is the first such study conducted in Mali since 2002 [17]. Our study supports the implementation of prophylactic HPV vaccination programs with vaccines against subtypes 16 and 18 in Mali in concordance with WHO recommendations [36] and provides information to guide future interventions for executing successful vaccination campaigns in West Africa. Our study shows that HPV vaccination is desired, and also confirms the need to engage male partners or heads of households in the decision-making process, regarding HPV vaccination. Urgent implementation of HPV vaccination in Mali will avert tens of thousands of deaths and is critically important for the wellbeing of Malian women and their families.

## Supporting information

S1 File An English translation of the standardized questionnaire used to collect general and medical information and willingness to participate (WTP) in an HPV vaccination program and vaccination decision-making capacity. This questionnaire was adapted from a pilot study published by our group in 2013 [18].
(DOCX)
