# Supplementary material for: Prevalence of HPV 16 and 18 and attitudes toward HPV vaccination trials in patients with cervical cancer in Mali
Source: PLoS One. 2017 Feb 23;12(2):e0172661. doi: 10.1371/journal.pone.0172661 (PMC5322926; doi:10.1371/journal.pone.0172661)
Supplement: S1 File — This questionnaire was adapted from a pilot study published by our group in 2013 [18]. (DOCX) [file pone.0172661.s001.docx]

Supplemental Data 1

**Survey Document (English translation)**

**I – Participant Information**

**Age:**

**1. Marital Status:**

Married

Married in a polygamous family

Single

Divorced

Widow

Pass

**2. How old were you when you got married?**

**3. Do you have children?**

If yes: How many children ho you have?

How old were you when you had your first child?

**4. Did you go to school?**

If yes: Until what grade?

K

5th

Middle School

High School

College

**5. Do you have an occupation?**

If yes, what is your occupation?

Agriculture

Saleswoman/shopkeeper

Administration

Nurse

Housewife

Cleaning lady

**II – Sexual life**

**6. How old were you at your first sexual intercourse?**

**7. How many sexual partners did you have before your marriage?**

**8. Do you use birth control?**

If yes, which one?

Pill

Injection

Other

**III - HPV and cervical cancer**

**9. How old were you when cervical cancer was diagnosed?**

**10. What gynecological exam did you have since the diagnosis?**

VIA

VILI

Biopsy

Colposcopy

HIV test and rhesus typing

**11. Are you under treatment?**

If yes, which one?

**12. Do you have other IST(s)?**

If yes, which one(s)?

GYN infections

Leuchorea

Prurit+Leuchorea

**IV - Vaccine**

**13. Have you been vaccinated in the past?**

If yes, which vaccine:

EVP (Extended Vaccination Program, includes diphteria, tetanus, whooping cough)

VAT (tetanus vaccine)

Meningitis

Yellow Fever

MMR

**14. If a vaccine against cervical cancer was available, to whom should it be administered?**

(Check one or more)

Adolescent females

Adolescent males

Women

Men

**15. If you were to get vaccinated, who would take the decision/give you permission?**

Father

Mother

Husband

Yourself

**16. Vaccination against cervical cancer is done by an injection in the arm**

Yes

No answer

**17. The vaccine against cervical cancer is already available in Europe and in the USA. Would you be willing to participate to a trial to obtain approval of this vaccine in Mali?**

**18. What would be the main reason to participate into a vaccine trial?**

Fight the disease / Fight cancer

For other women and generation of women

To have the vaccine available in Mali

**19. What would be the main reason not to participate into a vaccine trial?**

None

Afraid

Husband's authorization

Price

**20. I would get vaccinated/ I would vaccinate my child against cervical cancer if**

The vaccine was free

The vaccine cost less than

The price is reasonable

I will never get vaccinated
